# Ventilation control of road tunnels towards disturbance suppression

**DOI:** 10.1038/s41598-024-52816-8

**Published:** 2024-01-28

**Authors:** Yimeng Wang, Changxuan Zhou, Qitao Zhao, Ruihan Jia, Wei Wu

**Affiliations:** 1 School of Automobile, Chang’an University, Xi’an, 710064 China; 2 School of Electronics and Control Engineering, Chang’an University, Xi’an, 710064 China; 3 CCCC Second Highway Engineering Co., Ltd., Xi’an, 710065 China; 4 School of Automation, Northwestern Polytechnical University, Xi’an, 710129 China; 5 Autonomous Driving Center, SAIC Motor R&D Innovation Headquarter, Shanghai, 201804 China

**Keywords:** Civil engineering, Electrical and electronic engineering, Mechanical engineering

## Abstract

In recent years, research on ventilating tunnels has become increasingly important. However, the impact of external disturbances on ventilating systems has been largely ignored. To address this issue of frequent airflow fluctuations caused by external perturbations, which cannot be fully compensated using conventional control methods, this study proposes a perturbation-compensated ventilation control approach. A disturbance compensator is developed by incorporating the tunnel’s airflow velocity and the number of jet fan start-stop events as input parameters. By compensating for external disturbances, the disturbance to the system is reduced. The Simulink model of the tunnel controller was used for simulation experiments. The compensator demonstrated good tracking results in comparison experiments with different disturbances. The ventilation approach based on disturbance compensator is capable of regulating the fluctuation of CO concentration within a justifiable range compared to using PID control and ADRC. This not only improves the stability of the entire control system but also significantly prolongs the service life of the jet fan by reducing the frequency of start-stop cycles.

## Introduction

With the rapid urbanisation seen today, the inflow of people into cities has severely strained the transportation infrastructure^1^. Tunnels, as efficient transport facilities that can overcome obstacles, are effective in reducing travel distances and relieving traffic congestion in cities. Since tunnels are partially enclosed, maintaining an efficient ventilation system within them is vital. In the survey of motorway tunnels, Mohammad et al. discovered a significant increase in the concentration of CO and particulate matter in the compartment once cars entered tunnels that possessed poor ventilation systems^2^. Effective exhausting of the exhaust air in a semienclosed tunnel space is necessary to maintain a good tunnel environment. However, sudden changes in airflow can result from various factors, including air pressure differences, wind load, moving vehicles, chimney effect, amongst others^3^. Therefore, the tunnel ventilation system must integrate appropriate ventilation control methods to correct any disruptive effects on the tunnel environment.

In recent years, tunnel ventilation control system models and control methods have been extensively researched by scholars, resulting in several significant findings. Zhang et al. investigated the effects on natural ventilation and discussed control strategies for jet fans^4^. Hong Y has developed numerous enhanced PID control techniques to improve the practical use of PID control algorithms in the field of tunnelling^5^. Šulc and colleagues proposed an innovative ventilation control approach using an adaptive feedforward strategy^6^. The method mitigates deviations between mathematical models and actual data by compensating for them, and underwent validation within the Blanka Tunnel of Prague, Czech Republic. Ercüment Karakaş uses fuzzy control to significantly reduce power consumption while keeping pollution levels within acceptable limits^7^. Hrbček et al. demonstrated that the model predictive controller can effectively reduce the pollution value in the highway tunnel tube^8^. Peng et al. proposed an adaptive optimal control method based on MPC by investigating the dynamics of a road tunnel ventilation system^9^. The adaptive model predictive control calculates the optimal number of jet fans required to maintain pollutant concentration below the desired level in the road tunnel. Baeksuk Chu et al. optimized the design of fuzzy control using the stochastic global search method genetic algorithm (GA) for the conventional fuzzy control^10^. The authors minimized power consumption while maintaining control effectiveness of the controller, resulting in improved energy efficiency compared to the conventional control method. Liu et al. designed a tunnel ventilation intelligent inverter control (TVIC) system based on radial basis function neural network (RBF NN)^11^. The system allows for the determination of relationships between fan operation frequency and different pollutant concentrations, tunnel length, and temperature with the aid of the RBF neural network. The frequency of the fan is adaptively modified based on the construction environment in the tunnel. The experimental findings demonstrate that the system is dependable and efficient in enhancing the tunnel's environment and conserving energy. Euler-Rolle et al. proposed a method for controlling air flow velocity in tunnels under fire conditions^12^. The method involves feedback linearisation of the nonlinear air flow model and utilizing the resulting nonlinear relationship in the feedforward model of the jet fan's rotation for longitudinal ventilation control. This ensures that the air velocity within the tunnel is kept within a certain range. While the above approach enhances the ventilation control capacity, the model based ventilation method has some difficulties in dealing with external disturbances, and it is difficult to achieve the ideal control effect in the face of complex disturbances.

To address the perturbations and uncertainties in the model, Han J developed a ventilation control method that utilises Active Disturbance Rejection Control (ADRC)^13^. The ADRC control technique attains resistance to external disturbances by merging an observer with a nonlinear controller. It has attained extensive use in the domains of electric power^14^, autonomous driving^15^, and water resources^16^. Although ADRC offers the advantage of model independence, practical parameter tuning is a difficult and challenging task. Fuhrmann et al. in order to improve the disturbance attenuation of the tunnel ventilation system, a pressure drop observer was designed for observing disturbances with unknown inputs in the tunnel^17^. The control loop's disturbance compensation capability has been enhanced by factoring in perturbations in the control signal of the feed-forward section of the two-degree-of-freedom control plan. However, solely the alteration in airflow velocity was taken into account while the correlation between the jet fan and pollutant concentration was not tackled.

This paper focuses on the study of tunnel air dynamics and pollutant concentration modelling, proposing a ventilation control method that aims to suppress disturbances. The method involves analyzing the mathematical model of tunnel airflow and incorporating a disturbance compensator into the control model. The disturbance compensator tracks and compensates for disturbances to the controller, effectively reducing their impact on control performance. By adjusting the number of times the jet fans start and stop, the controller can consistently regulate the fluctuations in CO concentration within the tunnel.

The paper is divided into 5 parts. In “Mathematical model of the tunnel” section, we established the mathematical models of the tunnel, tunnel air flow and tunnel ventilation. In “The disturbance compensator design” section, the design and model construction of a disturbance compensator is proposed. In “Evaluation” section, the experimental results are shown. The conclusion is given in “Conclusions” section.

## Mathematical model of the tunnel

**Figure 1:**
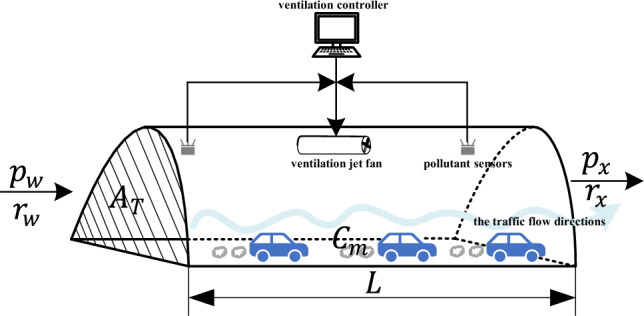
The structure diagram of tunnel system.

To investigate the tunnel ventilation control problem, we construct a schematic diagram of the tunnel ventilation system as shown in Fig. 1. The diagram includes the pollutant sensors, ventilation jet fans, the vehicle travelling in the tunnel and the controller to control the ventilation jet fan.

Where $p_{w}$ and $p_{x}$ represent the pressure at the inlet and outlet of the tunnel respectively. The $r_{w}$ and $r_{x}$ represent the air velocities at the entrance and exit of the tunnel, respectively. The $A_{T}$ denotes the longitudinal cross-sectional area of the tunnel, $L$ denotes the total length of the tunnel, and $C_{m}$ denotes the concentration of pollutants in the tunnel.

**Figure 2:**
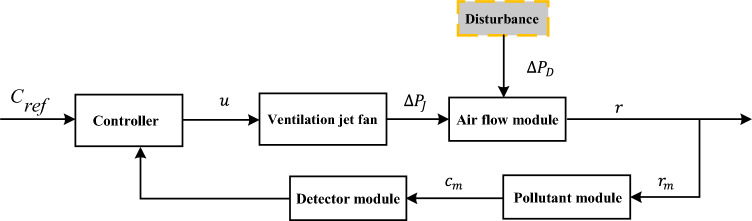
Structure of the system model.

The modeler is not required to directly address smoke or thermal diffusion. Instead, they only need to take into account the average velocity of the airflow within the tunnel. Furthermore, the model is built on the assumption that the air within the tunnel is a stable, continuous, and incompressible fluid. To create a comprehensive model of the tunnel control system, the tunnel schematic is examined and the system model is depicted in Fig. 2.

In the structural diagram of the system model, it concludes controller, ventilation jet fan, airflow module, and pollutant module. Where $C_{ref}$ denotes the pollutant concentration limit in the tunnel, $u$ denotes the control variable input to the fan model, $\Delta P_{J}$ denotes the pressure change generated in the tunnel during the fan control process, $\Delta P_{D}$ denotes the disturbance in the tunnel, $r_{m}$ denotes the air flow velocity in the tunnel under the action of the controller.

### Tunnel pollutant concentration model

According to the law of conservation of mass, the concentration of pollutants in the tunnel can be measured^18^:

1 $$\frac{c(t + \delta t) - c(t)}{{\delta t}} = \frac{1}{{LA_{T} }}\left( {C_{veh} (t) - M_{e} r(t)c(t)} \right)$$

where $C_{veh} (t)$ represents the pollutants released by vehicles per unit of time, $M_{e}$ represents the cross-sectional area of the pollutant outlet in the tunnel, $c(t)$ represents the concentration of pollutants per unit time, $r(t)$ represents the wind speed per unit time within the tunnel. As $t$ tends to zero, Eq. (1) can be rewritten as^9^:

2 $$c\dot{(}t) = \frac{1}{{LA_{T} }}\left( {C_{veh} (t) - M_{e} r(t)c(t)} \right)$$

From Eq. (2), the pollutant concentration $c(t)$ in the tunnel can be reduced by adjustment of the wind speed $r(t)$ in the tunnel, so that the pollutant concentration $c(t)$ will be lower than the reference value $C_{ref}$. This ensures the tunnel's air quality remains within safety parameters.

### Tunnel air flow model

According to Bernoulli's equation for an ideal fluid, the difference in pressure between any two points in the tunnel can be calculated as follows^19^:

3 $$\frac{1}{2}\rho \left( {r_{w}^{2} - r_{x}^{2} } \right) = p_{w} - p_{x}$$

where $\rho$ is the tunnel air density.

When the pressure in the tunnel changes, Eq. (3) is converted to^20^:

4 $$\frac{1}{2}\rho \left( {r_{w}^{2} - r_{x}^{2} } \right) - \rho \int_{0}^{L} {\frac{\partial r(s,t)}{{\partial t}}} ds + \Delta p(t) = p_{w} - p_{x}$$

where the integrated term is pressure change due to non-constant flow and $\Delta p(t)$ is the total pressure change in the tunnel due to pressure loss and pressure gain.

It is assumed that the entrance and exit pressures of the tunnel are identical, i.e., $p_{w} = p_{x}$, while the airflow in the tunnel remains steady, i.e., $r_{w} = r_{x} = r_{m}$. Therefore, the Eq. (4) can be simplified as follows^20^:

5 $$\rho L\frac{dr(t)}{{dt}} = \Delta p(t)$$

The total pressure change $\Delta p(t)$ within the tunnel is:

6 $$\Delta p(t) = \Delta p_{F} (t) + \Delta p_{J} (t) + \Delta p_{a} (t) + \Delta p_{p} (t) + \Delta p_{w} (t) + \Delta p_{M} (t)$$

where $\Delta p_{F} (t)$ denotes the pressure variation in the tunnel caused by air friction, which is the resistance to local ventilation, $\Delta p_{J} (t)$ denotes the pressure variation in the tunnel caused by jet fans. Others, $\Delta p_{a} (t)$, $\Delta p_{p} (t)$, $\Delta p_{w} (t)$ and $\Delta p_{M} (t)$ correspond to pressure variations due to reduction of the area, vehicle piston effect, wind entering the tunnel, and meteorological pressure discrepancies inside and outside the tunnel, respectively. Due to the involvement of several factors in the computation of the piston effect in tunnel vehicles, such as vehicle velocity, tunnel dimensions, and tunnel characteristics, this paper concentrates on the suppression of tunnel ventilation disturbances and the piston effect is not discussed in detail here.

Considering that $\Delta p_{F} (t)$ and $\Delta p_{J} (t)$ dominate the pressure changes in the tunnel. $\Delta p_{a} (t)$, $\Delta p_{p} (t)$, $\Delta p_{w} (t)$, and $\Delta p_{M} (t)$ have small values in the tunnel. Therefore, the tunnel model does not consider secondary pressure effects, but considers secondary pressure variations as external disturbances to the system, and corrects their deviations using a compensator. Thus rewriting (5) and (6) as:

7 $$\rho L\frac{dr(t)}{{dt}} = \Delta p_{F} (t) + \Delta p_{J} (t)$$

The pressure variation within the tunnel resulting from the operation of the jet fan can be modelled as follows^9^:

8 $$\Delta p_{J} (t) = k_{JF} N(t)\left( {V_{JF} - r(t)} \right)$$

where $k_{JF} = \frac{{\eta \rho A_{JF} V_{JF} }}{{A_{T} }}$, $V_{JF}$ is the nominal output airflow velocity of the jet fan, $\eta$ is the jet fan efficiency, $N(t)$ is the number of fans operating simultaneously in the tunnel, and $A_{JF}$ is the cross-sectional area of the fan outlet.

The pressure change in the tunnel caused by air friction can be formulated as follows^17^:

9 $$\Delta p_{F} (t) = k_{Fric} r(t)\left| {r(t)} \right|$$

where $k_{Fric}$ represents the coefficient of air friction.

### Tunnel ventilation model

Combined with the analysis of the above models, the overall ventilation model of the road tunnel is constructed as follows:

10 $$\left\{ {\begin{array}{*{20}l} {\mathop {r(t)}\limits^{ \cdot } = \frac{{k_{JF} V_{JF} }}{\rho L}N(t) - \frac{{k_{JF} }}{\rho L}N(t)r(t) + \frac{{k_{Fric} }}{\rho L}r(t)\left| {r(t)} \right|} \hfill \\ {\mathop {c(t)}\limits^{ \cdot } = - \frac{{M_{e} }}{{LA_{T} }}r(t)c(t) + \frac{1}{{LA_{T} }}C_{veh} (t)} \hfill \\ \end{array} } \right.$$

Let $x_{1} (t) = r(t)$, $x_{2} (t) = c(t)$, the input is $u(t) = N(t)$ and the output is $y(t) = c(t)$. The change in pollution caused by the vehicles travelling in the tunnel can be further rewritten as (10):

11 $$\left\{ {\begin{array}{*{20}l} {\mathop {x_{1} (t)}\limits^{ \cdot } = \frac{{k_{JF} V_{JF} }}{\rho L}u(t) - \frac{{k_{JF} }}{\rho L}u(t)x_{1} (t) + \frac{{k_{Fric} }}{\rho L}x_{1} (t)\left| {x_{1} (t)} \right|} \hfill \\ {\mathop {x_{2} (t)}\limits^{ \cdot } = - \frac{{M_{e} }}{LM}x_{1} (t)x_{2} (t) + \frac{1}{LM}C_{veh} (t)} \hfill \\ \end{array} } \right.$$

12 $$y(t) = x_{2} (t)$$

## The disturbance compensator design

**Figure 3:**
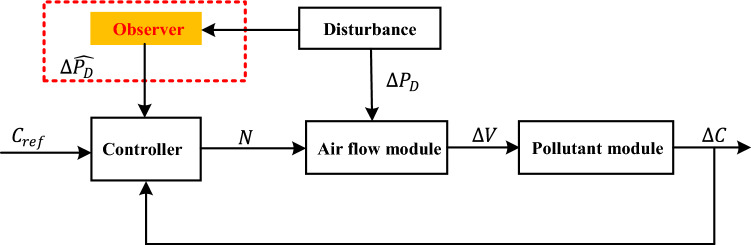
The compensator design framework.

Due to the presence of random disturbances within the tunnel, the mathematical model of tunnel ventilation, formulated by Eqs. (11) and (12) in the tunnel access risk control module depicted in Fig. 3, does not yield satisfactory control results. In order to enhance system performance and ensure that the concentration of CO remains stable within an acceptable range, we have conducted a comprehensive analysis of the airflow state space described by the mathematical model (11). Furthermore, we have devised a disturbance compensator to mitigate the impact of errors on tunnel ventilation control.

If there is no compensator design in place, an external disturbance $$\Delta p_{D}$$ will be applied to the system, resulting in the generation of an extra disturbing airflow $$\Delta V$$ by the air-flow module. This will cause fluctuations $$\Delta C$$ in the pollutant module. As disturbances can disrupt the controller, it may not be able to provide sufficient control. That is to indicate that the current manipulated variable $$N$$ is not sufficient to reduce the pollutant concentration in the tunnel to within the appropriate range. Thus, we present a compensator design that adeptly tracks external disturbances and integrates the compensation of observed system perturbations $$\Delta \overset{\lower0.5em\hbox{$\smash{\scriptscriptstyle\frown}$}}{p}_{D}$$ into the controller, ultimately aiming to boost the efficacy of the control mechanism.

### Analysis of tunnel air flow state space

When unknown disturbances $\Delta p_{D}$ are present in the system, model (7) can be adjusted:

13 $$\frac{dr(t)}{{dt}} = \frac{1}{\rho L}\left( {\Delta p_{Fric} (t) + \Delta p_{JF} (t){ + }\Delta p_{D} (t)} \right)$$

Further changes:

14 $$r\dot{(}t) = \frac{{k_{JF} }}{\rho L}N(t)\left( {V_{JF} - r(t)} \right) - \frac{{k_{Fric} }}{\rho L}r(t)\left| {r(t)} \right| + \frac{1}{\rho L}\Delta p_{D} (t)$$

Thus, the observed tunnel state space is a continuous-time nonlinear system with multiple inputs and single output^21^:

15 $$\left\{ {\begin{array}{*{20}l} {{\varvec{z}}_{1} \dot{(}{\varvec{t}}) = f({\varvec{z}}({\varvec{t}}),{\varvec{n}}({\varvec{t}}))} \hfill \\ {{\varvec{y}}({\varvec{t}}) = g({\varvec{z}}({\varvec{t}}))} \hfill \\ \end{array} } \right.$$

where the state vector ${\varvec{z}}({\varvec{t}})$ is defined as:

16 $${\varvec{z}}({\varvec{t}}) = \left[ \begin{gathered} \hat{r}(t) \\ r(t) \\ \Delta p_{D} (t) \\ \end{gathered} \right]$$

Here, the $z_{1} (t) = \hat{r}(t)$ is the airflow velocity calculated by the model, the output is $y(t) = r(t)$ and control variable is $n(t) = N(t)$.

Since there is no prior information about $\Delta p_{D}$, it is not possible to calculate the perturbation of the whole system in advance. So we begin by assuming constancy, whereby its derivative with respect to time is 0, i.e. $\Delta \dot{p}_{D} = 0$. By combining Eqs. (14) and (16), the state space equations for the tunnel can be structured as follows:

17 $${\varvec{z}}\dot{(}{\varvec{t}}) = \left[ \begin{gathered} \frac{{k_{JF} }}{\rho L}n(t)\left( {V_{JF} - z_{1} (t)} \right) \\ - \frac{{k_{Fric} }}{\rho L}z_{1} (t)\left| {z_{1} (t)} \right| + \frac{{z_{3} (t)}}{\rho L}\frac{1}{{\tau_{m} }}\left( {z_{1} (t) - z_{2} (t)} \right) \\ 0 \\ \end{gathered} \right]$$

where system output $y(t)$ is the air flow in the tunnel:

18 $$y(t) = g(z) = z_{2} (t)$$

To support the design of the compensator, we organize the state space and further decompose Eq. (17) into a linear part and a nonlinear part of the form:

19 $$\begin{aligned} {\varvec{z}}\dot{(}{\varvec{t}}) & = \left[ {\begin{array}{*{20}c} 0 & 0 & 0 \\ {\frac{1}{{\tau_{m} }}} & { - \frac{1}{{\tau_{m} }}} & {\frac{1}{\rho L}} \\ 0 & 0 & 0 \\ \end{array} } \right]z + \left[ \begin{gathered} \frac{{k_{JF} }}{\rho L}n(t)\left( {V_{JF} - z_{1} (t)} \right) \\ - \frac{{k_{Fric} }}{\rho L}z_{1} (t)\left| {z_{1} (t)} \right| \\ 0 \\ \end{gathered} \right] \\ & = A{\varvec{x}} + \delta \left( {z_{1} ,n} \right) \\ \end{aligned}$$

where

20 $$\delta \left( {z_{1} ,n} \right) = \left[ \begin{gathered} \sigma \left( {z_{1} ,n} \right) \\ 0 \\ 0 \\ \end{gathered} \right]$$

After introducing the perturbation, the complex state space problem is decomposed into a combined problem consisting of a linear and a nonlinear part using state space analysis and equivalent transformation for the tunnel. For the linear part, we use the PID control method to solve. While dealing with the nonlinear part can be complex, we simplify a multivariate nonlinear problem to a nonlinear problem related to the state variable $z_{1}$ through matrix decomposition treatment. As shown in (20), thus we have completed the simplification based on the airflow state space.

### The perturbation compensator model

**Figure 4:**
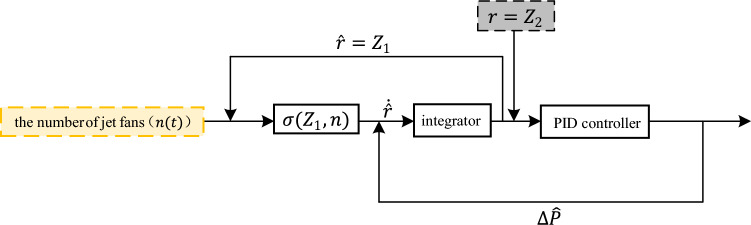
Structure diagram of disturbance compensator.

To reduce the influence of disturbances on the control performance of the controller, we design the disturbance compensator based on the nonlinear part $\delta (z_{1} ,n)$ of the state space of the tunnel aerodynamic model. The overall structure of the compensator is shown in Fig. 4.

The compensator is adjusted by the PID controller so that the difference between its estimated and calculated values is equal to the disturbance introduced into the system. Euler-Rolle et al.^22^ demonstrated that optimisation of a model's parameters can lead to improved accuracy. As a result, a tunnel model is built that adequately describes the controlled system, i.e., deviations between the controlled object and the model of the controlled object are only due to external perturbations. Therefore, the control variables in the disturbance compensator are continuously adjusted by the PID controller to reduce the influence of the disturbance and achieve a more accurate control model.

In Fig. 4, the non-linear component $\sigma (z_{1} ,n)$ can be depicted as follows:

21 $$\sigma \left( {z_{1} ,n} \right) = \Delta p_{F} (t) + \Delta p_{J} (t)$$

The estimation of the tunnel air velocity is achieved by integrating the inputs $n(t)$ and the estimated disturbance $\Delta p_{D} (t)$, resulting in a continuous convergence to the external perturbation. This estimate is then compared to the calculated air velocity of the model, resulting in accurate tracking performance.

**Figure 5:**
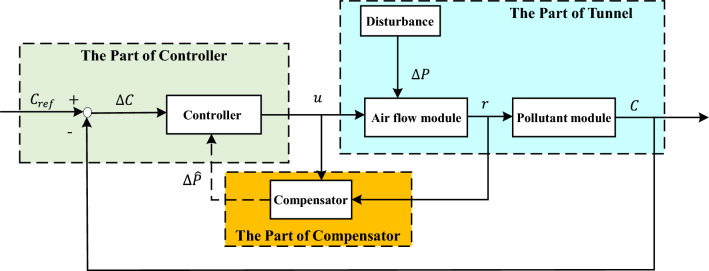
Tunnel ventilation design block diagram.

The aforementioned compensator is incorporated into the road tunnel ventilation model as illustrated in Fig. 5. This model is constructed of three main elements: the tunnel model, the controller, and the disturbance compensator. Persistent disturbances infiltrate the control process via external inputs and can exert a significant influence over tunnel ventilation effectiveness once they cross a certain boundary. When pollutant concentrations within the tunnel elevate beyond recommended limits, steering the ventilation system effectively becomes a formidable task.

By incorporating the compensator model, it becomes possible to monitor and track the disturbances occurring in the tunnel over time. These tracked disturbances can then be utilized by the controller to compensate for their effects. This compensation helps mitigate the overall impact of the disturbances on the control system as a whole, ensuring that pollutant concentration levels in the tunnel remain within acceptable ranges.

## Evaluation

To verify the control strategy proposed in this paper, we build a tunnel ventilation simulation model based on Matlab/Simulink to verify the simulation example of a road tunnel ventilation control. For the parameters of the tunnel ventilation model^9^, it is shown in Table 1.

**Table 1:** Ventilation system parameters.

Parameter	Value
The length of tunnel $L$	3300 m
The design speed of tunnel	80 km/h
The number of tunnel jet fans	8
The air density $\rho$	1.23 kg/m^3^
The cross-section area of tunnel $A_{T}$	65.65 m
The cross-sectional area of pollutant outlet $M_{e}$	88.35 m^2^
The cross-sectional area of jet fans $A_{JF}$	0.83 m^2^
The nominal flow rate of jet fans $V_{JF}$	27 m/s
The efficiency of jet fans $\eta$	0.85
The coefficient of air friction $k_{Fric}$	0.8

In this simulation, we set the reference value for CO concentration at 60 ppm. The simulation lasted for 6000 s, with a simulation step of 1 s. In order to assess the control performance of the disturbance compensator-based approach, we conducted a comprehensive analysis of the ventilation control impact, considering the presence of disturbance signals. The disturbance compensator-based approach is evaluated by comparing it with the traditional PID method and the ADRC method described in reference 13. We performed simulation experiments under three different scenarios: one with a constant value disturbance, another with a non-constant disturbance, and the last one with random disturbances. By simulating these various types of disturbances, we aimed to validate the reliability of the disturbance compensator-based approach.

### Simulation experiment under the influence of constant value disturbance

**Figure 6:**
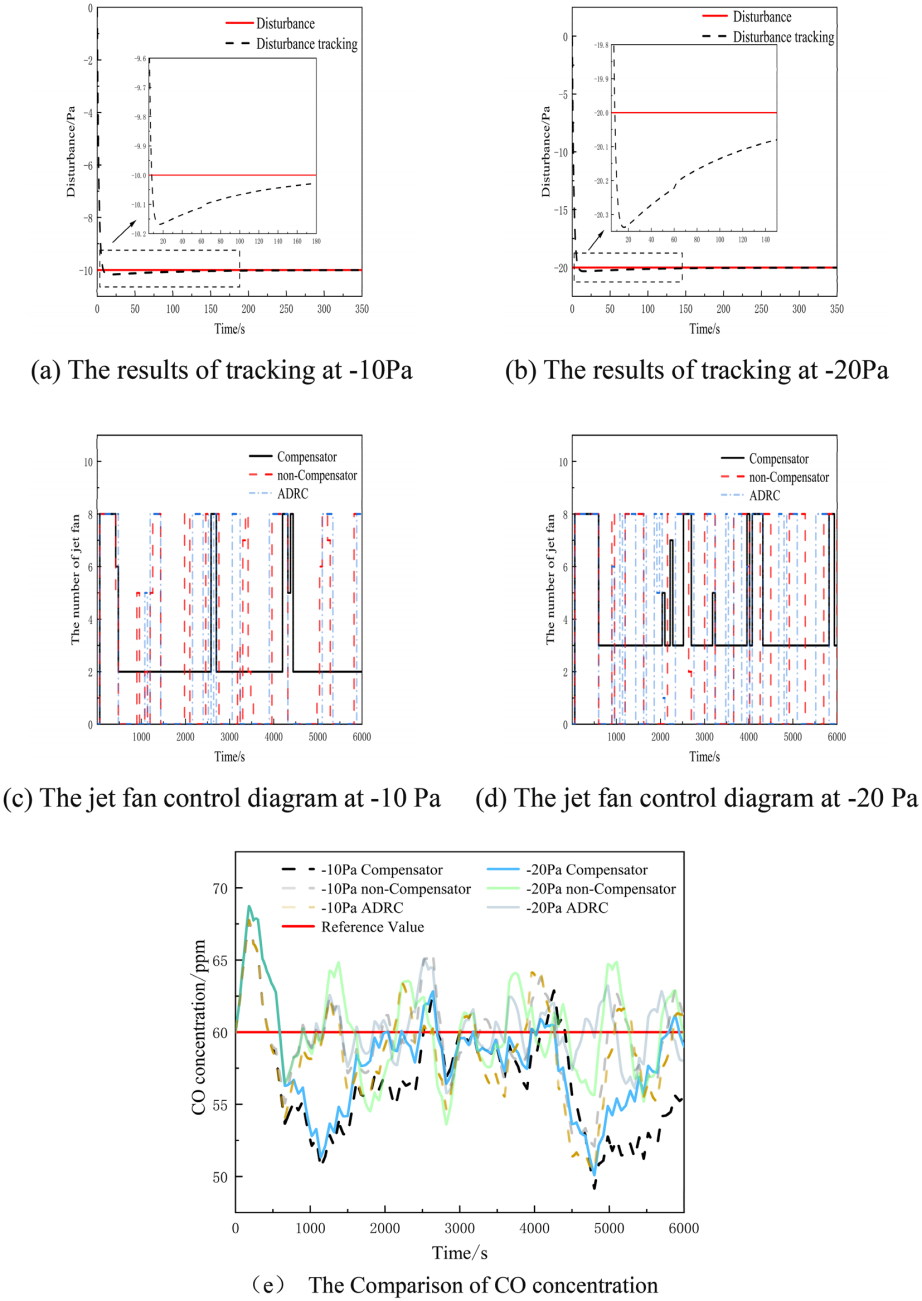
Figure of experimental results under the influence of constant disturbance.

To verify the effectiveness of compensator-based control, we simulate tunnel ventilation control with − 10 Pa and − 20 Pa as constant external disturbances. The results of the simulation are shown in Fig. 6.

Figure 6a and b illustrate how the perturbation compensator effectively tracks disturbances in the control system. In Fig. 6a, the tracking value reaches its maximum of − 10.1686 Pa at 16 s, with an error peaking at 1.686%. Subsequently, the error gradually decreases, and the tracking disturbance closely aligns with the disturbance curve at 300 s. Similarly, Fig. 6b shows the tracking value reaching its maximum of − 20.3371 Pa at the 17th second, with an error peaking at 1.6855%. The error then gradually decreases, and the tracking disturbance closely matches the perturbation curve at 300 s. These diagrams demonstrate that the disturbance compensator achieves accurate tracking in both constant disturbance scenarios.

Figure 6c and d further confirm the stability of the compensator-based ventilation control method over the PID control method and ADRC method. Regardless of the effect of − 10 Pa and − 20 Pa perturbations, the compensator-based control method exhibits fewer fluctuations in fan activations. By compensating for disturbances through the compensator, the method reduces the impact of disturbances on the concentration, minimizes pollutant concentration variations, and ensures more stable fan activations.

Figure 6e illustrates the comparison of the distribution of pollutant concentration in the tunnel under two different control system inputs: − 10 Pa and − 20 Pa perturbation. Initially, the concentration of CO experiences a significant overshoot, reaching a maximum of 67.77683 ppm when the disturbance is − 10 Pa. Conversely, when the disturbance is − 20 Pa, the CO concentration reaches a maximum overshoot of 68.73708 ppm.

During the first 600 s, the non-correlated detectors have no effect on the CO concentration. This is due to the limited number of tunnel fans and the hysteresis in gas concentration changes, which hinder an immediate reduction of the CO concentration in the initial moments. At 660 s, it becomes evident that the compensator-based control method is still capable of maintaining the CO concentration in a decreasing state. Meanwhile, the CO concentration under the no-compensator control starts to increase and quickly exceeds the reference value. Specifically, under the influence of the − 20 Pa disturbance, the CO concentration reaches an overshoot of 64.46 ppm. While the ADRC method is more effective at − 10 Pa, it performs poorly when the disturbance is − 20 Pa. At this disturbance, the CO concentration reaches 68.729 ppm at 188 s.

Due to the cumulative effect of the disturbance on the system, the compensator-based control leads to slower changes in the CO concentration compared to the PID control. As the simulation progresses, the compensator-based controller successfully keeps the CO concentration within a reasonable range, despite a tendency to increase. The compensator's capability of tracking and compensating for disturbances within the controller mitigates the impact of CO concentration fluctuations on the turbine's control.

Conversely, the controller without a compensator displays fluctuations in CO concentration when subjected to disturbances. These fluctuations become more pronounced as the disturbance increases, as a result of inadequate turbine control, ultimately preventing the CO concentration from fluctuating within a suitable range. In correspondence with the ADRC method, in cases where the intensity of the external disturbance surpasses the control capability range of the ADRC, even if the ADRC is functioning optimally, it cannot fully eliminate the impact of the disturbance on the CO concentration, leading to insufficient control performance. Therefore, the compensator-based method proves to be more effective in controlling the system under constant value disturbances.

### Simulation experiments under the influence of non-constant disturbance

During tunnel operations, disturbances are characterized by strong randomness and rarely occur as fixed disturbances. Therefore, the external random disturbances are modeled using a multiband function to represent the system's disturbance through frequent and variable functional relationships. The equation for the disturbance function is as follows:

22 $$f(t) = \left\{ {\begin{array}{*{20}l} { - \frac{t}{100},} \hfill & {\quad t \le 1000\,{\text{s}}} \hfill \\ { - \frac{{t^{2} }}{{10^{6} }} - 9,} \hfill & {\quad 1000 < t \le 3000\,{\text{s}}} \hfill \\ { - \frac{t}{150} - 6,} \hfill & {\quad 3000 < t \le 5000\,{\text{s}}} \hfill \\ {\frac{t}{200} - 51,} \hfill & {\quad \, t > 5000\,{\text{s}}} \hfill \\ \end{array} } \right.$$

**Figure 7:**
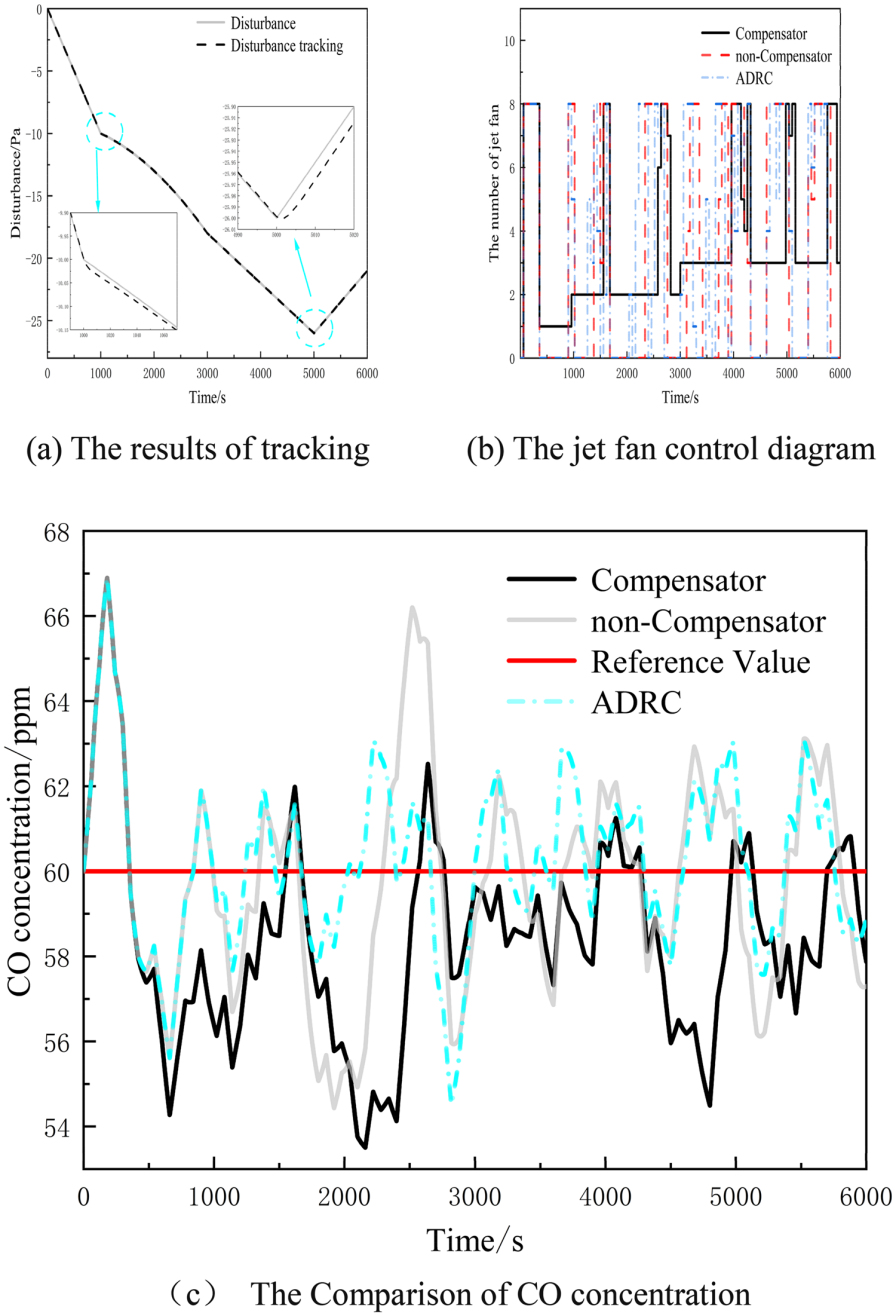
Figure of experimental results under the influence of non-constant disturbance.

The compensator-based method for controlling ventilation has been confirmed in this perturbation case, with the experimental results shown in Fig. 7.

Figure 7a demonstrates the results of tracking compensator disturbances in the presence of a non-constant disturbance. The figure illustrates that at 1000 s and 5000 s, the disturbance experiences sudden changes, resulting in reduced observation performance and larger tracking errors in the control system. However, the maximum tracking error remains below 2% and is reduced to 1% within 300 s. The PID controller effectively monitors the air flow rate, accounting for slight hysteresis in the actual rate, resulting in an error within an acceptable range of less than 2%. Thus, despite the presence of non-stationary disturbances, the compensator is still able to achieve satisfactory tracking results.

Figure 7c displays the changes in CO concentration within the tunnel when subjected to a non-constant perturbation. Due to a limited number of fans, the simulation reveals a maximum overshoot in CO concentration at 180 s, reaching 66.89493 ppm, which significantly exceeds the reference value. As the simulation progresses, the performance of the PID control diminishes, causing the CO concentration to frequently surpass the reference value. At 2520 s, the CO concentration reaches 66.20176 ppm. In contrast, the compensator-based control method effectively regulates the CO concentration, keeping it within an appropriate range. At 2640 s, the maximum recorded CO concentration is only 62.52563 ppm, representing a decrease of 6.531586%. Compared to a constant disturbance, the non-constant disturbance presents a greater challenge for the PID control, as the frequently changing disturbance causes constant fluctuations in CO concentration, deviating from the reference value and requiring continuous adjustments by the controller, thus increasing the load on the fan. The ADRC method is more effective than PID control under non-constant disturbances, but slightly less effective than the compensator. The compensator-based PID control compensates for the disturbance to the controller via the compensator, reducing CO concentration fluctuations, stabilizing the controlled variable, minimizing the frequency of fan start-up changes, and achieving a stable control effect.

### Simulation experiments under the influence of random disturbance

**Figure 8:**
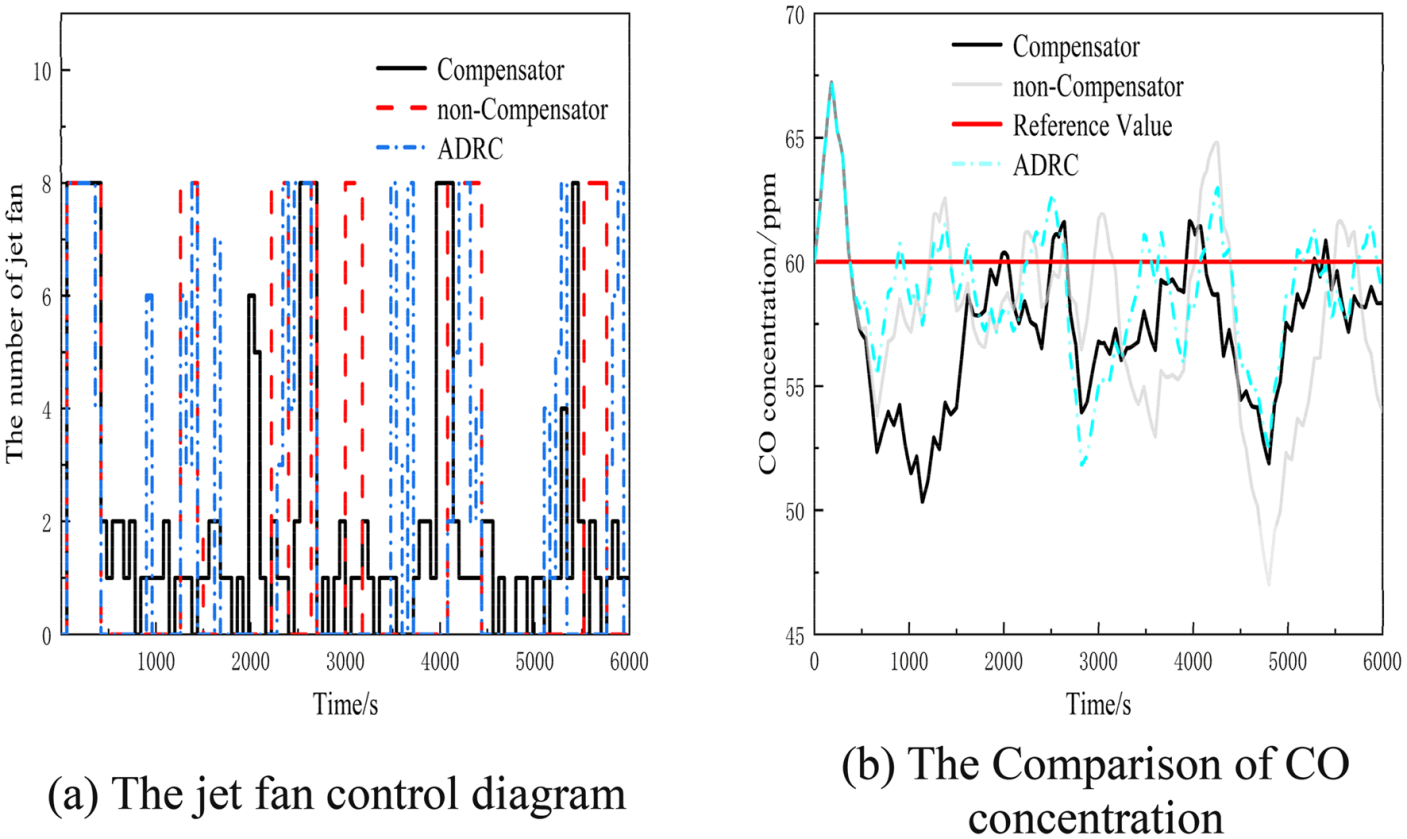
Figure of experimental results under the influence of random disturbance.

In order to evaluate the effectiveness of the compensator-based ventilation control method in mitigating disturbances under challenging conditions, this experiment employs white noise ranging from − 15 to 5 Pa to simulate their influence on the system^23^. The outcomes of the experiment are depicted in Fig. 8.

The nature of white noise makes it difficult to quantitatively analyze the tracking effect of the compensator-based control method. Therefore, this analysis does not specifically address the tracking effect. It is important to note that the random disturbances depicted by white noise in the experiment are not realistic and extreme environmental changes are unlikely to occur in reality. However, the main purpose of this experiment is to verify the stability and effectiveness of the tracking-based control method.

In the worst-case scenario, where the disturbance continuously fluctuates violently, the experiment aims to validate the robustness and feasibility of the tracking-based control method. If the controller can continue to function correctly and maintain a relatively stable control effect in this situation, it indicates that the method is robust in the presence of disturbances.

Figure 8b demonstrates the effectiveness of both the compensator-based, ADRC method and PID control method in regulating CO concentration. Initially, due to control hysteresis, the CO concentration gradually increases. However, after 580 s, the compensator-based control method achieves slightly more stable fluctuations in CO concentration compared to the PID method and ADRC method.

The compensator-based control method effectively mitigates the CO concentration with a peak value of 61.645 ppm, while the PID method has a peak value of 64.814 ppm and the ADRC method has a peak value of 62.898 ppm. Furthermore, the compensator-based control method maintains smoother fluctuations in CO concentration compared to the PID method and the ADRC method, which exhibits higher fluctuations and overshooting when faced with random disturbances. By compensating for disturbances, the compensator-based control method minimizes fluctuations in CO concentration and reduces overshoot.

In conclusion, the compensator-based ventilation control method demonstrates stability and effectiveness in mitigating disturbances under challenging conditions, as verified by the experiment.

### The controller analysis of stability and energy efficiency

In order to analyse the control effect of the controller for CO concentration, we analyse the control effect by using the average CO concentration in the tunnel (average CO concentration), the variance of the CO concentration (variance), the maximum overshoot after stabilisation (maximum overshoot), the area surrounded by the CO concentration exceeding the reference value (overshoot area), and the proportion of time that the CO concentration exceeds the reference value (overshoot proportion) as the metrics for evaluating the stability of the system. The statistical results of our data using Figs. 6e, 7c, and 8b are shown in Table 2.

**Table 2:** Analysis table of controller’s results.

	Constant disturbance		Non-constant disturbance	Random disturbance
	− 10 Pa	− 20 Pa		
Average CO concentration (ppm)				
PID	59.3757	60.1675	59.7991	57.8076
ADRC	58.5768	60.3990	60.1799	58.7717
Compensator	56.4904	58.0261	58.3785	57.3820
Reduction (ppm)				
PID	2.8853	2.1414	1.4206	0.4256
ADRC	2.0864	2.3729	1.8014	1.3897
Variance				
PID	8.8386	9.6505	6.7792	14.0347
ADRC	11.2750	5.8670	3.8356	7.0860
Compensator	14.0077	12.4932	5.4087	9.4371
Reduction (ppm)				
PID	− 5.1691	− 2.8427	1.3705	4.5976
ADRC	− 2.7327	− 6.6262	− 1.5731	− 2.3511
Maximum overshoot				
PID	65.48	64.85	66.2	64.76
ADRC	64.21	65.09	63.02	62.75
Compensator	62.88	62.28	62.51	61.62
Reduction (ppm)				
PID	2.6	2.57	3.69	3.14
ADRC	1.33	2.81	0.51	1.13
Overshoot area				
PID	5175.1980	8154.8066	5723.7980	3597.8475
ADRC	4298.2140	6609.2530	5172.0690	2965.1940
Compensator	2362.4084	3481.1875	2042.3108	1941.3998
Reduction				
PID	2812.7896 (54.35%)	4673.6191 (57.31%)	3681.4872 (64.32%)	1656.4477 (46.04%)
ADRC	1935.8056 (45.04%)	3128.0655 (47.33%)	3129.7582 (60.51%)	1023.7942 (52.73%)
Overshoot proportion				
PID	0.33949	0.5102	0.4954	0.2756
ADRC	0.3383	0.5261	0.5381	0.3454
Compensator	0.1550	0.2141	0.2315	0.1530
Reduction				
PID	0.18449	0.2961	0.2639	0.1226
ADRC	0.1833	0.3120	0.3066	0.1924

Table 2 demonstrates the superiority of the compensator-based ventilation control method over the PID method and the ADRC method in terms of regulating the average concentration of CO and minimizing overshoot. Notably, the compensator-based approach achieves a lower average CO concentration, as well as smaller overshoot ratios and maximum overshoot values. When exposed to random disturbances, the compensator-based ventilation control method exhibits a comparable average CO concentration to PID control and the ADRC method, yet achieves a 46.04%, 52.73% reduction in overshoot area and a 12.26%, 19.24% reduction in overshoot proportion. Regarding the evaluation metric of variance in Table 2, although the compensator-based ventilation method demonstrates improved control during customized perturbations, the necessity of compensating for observed perturbations in the controller introduces greater fluctuations and results in higher variance with fixed value perturbations. In contrast, the control performance of the PID method deteriorates with the introduction of disturbances, leading to increased overshooting, further highlighting the superior control achieved by the compensator-based ventilation method.

Table 2 presents results that clearly demonstrate the superiority of the compensator-based ventilation control approach over the conventional PID and ADRC methods in terms of regulating the average CO concentration and minimizing overshoot. The compensator-based approach achieved a significantly lower average CO concentration, as well as smaller overshoot ratios and maximum overshoot values. When random disturbances were introduced, the compensator-based ventilation control method showed comparable average CO concentration to the PID and ADRC methods, but with a remarkable reduction of 46.04% and 52.73% in overshoot area, as well as a reduction of 12.26% and 19.24% in overshoot proportion. Although Table 2 suggests that the compensator-based ventilation method may exhibit higher fluctuations due to compensating for observed perturbations, it is still evident that this approach offers improved control during customized perturbations compared to the PID and ADRC methods.

The paper also highlights the potential for energy savings through the control method, which involves regulating the frequency of turbine start and stop events, as well as monitoring and managing the turbine's total operating time. Notably, the control performance of the compensator-based ventilation method is not significantly affected by random disturbances, as shown in Table 3, which demonstrates the total fan running time and frequency of start-stop events during the simulation period of 6000 s, as illustrated in Figs. 6c, d and 7b.

**Table 3:** Analysis table of fan operation results.

	Constant disturbance		Non-constant disturbance
	− 10 Pa	− 20 Pa	
PID			
Starting and stopping frequency of the jet fan	140	144	156
Total running time (min)	268	402	345
ADRC			
Starting and stopping frequency of the jet fan	146	238	218
Total running time (min)	269	386	335
Compensator			
Starting and stopping frequency of the jet fan	44	69	81
Total running time (min)	271	400	353

Table 3 clearly demonstrates that the compensator-based ventilation control method outperforms the PID control and ADRC method in regulating the frequency of fan start-stop cycles, resulting in a significant reduction in these cycles and a longer lifespan for the jet fan. The primary reason behind this improvement is that the compensator-based method tracks disruptions and compensates for them within the controller, thereby reducing the overall interference of disruptions on the control system. As a result, the magnitude of CO concentration fluctuations is reduced, leading to a more stable control value and a lower frequency of fan start-stop events.

The compensator-based ventilation control method requires more fan running time compared to the PID control and ADRC method in order to effectively compensate for disturbances and provide additional control volume for enhanced performance. This increased fan running time results in improved stability as perturbations escalate, ultimately reducing the overall runtime of the turbine. In contrast, the traditional control method places a burden on the turbine by diminishing its control capability and increasing its total running time. Consequently, the compensation-based control method experiences a relative decrease in control time.

Hence, when compared to the PID control method and ADRC method, the compensator-based control method proves to be capable of effectively reducing the frequency of fan start-stop events, thus extending the service life of the jet fan and providing energy-saving benefits.

## Conclusions

This paper addresses the problem of tunnel ventilation control under various disturbances and considers tracking compensation of the disturbances to improve the stability of the ventilation control system. A disturbance compensator based ventilation control method is designed to achieve the disturbance suppression capability. Simulation experiments are carried out by modelling constant, non-constant and random perturbations. The results show that the compensator can track the disturbance faster. The compensator-based ventilation method can reduce the overshoot of the PID control method and make the ventilation control system more stable. Meanwhile, it can effectively reduce the start-stop frequency of the fan and prolong the service life of the fan.

As the compensator-based control methodology in this paper fulfils the control objective by manipulating the number of fan starts and stops, there exists a certain degree of energy wastage. Accordingly, exact control in partnership with frequency conversion control is a subsequent research focus.

## Data Availability

The datasets used and/or analysed during the current study available from the corresponding author on reasonable request.
